# Account of Diseases of the Eye, as They Prevail in Some Parts of India; with the Results of the Operations for Their Removal

**Published:** 1826-05

**Authors:** George Richmond

**Affiliations:** Assistant Surgeon 4th Light Dragoons.


**Art. VII.—*Account of Diseases of the Eye, as they prevail in some Parts of India; with the Results of the Operations for their Removal.*** By George Richmond, Assistant Surgeon 4th Light Dragoons.*

***Abstract of Diseases of the Eye, treated at Poonah by Surgical Operations, from the 6th of May to the 12th of December*, 1824.**

| *Diseases.* | *Restored to good Sight by Operation* | *Restored to good Sight by Operation; but, by the imprudence of the Patient, Inflammation was brought on, which destroyed the Eyes.* | *Total Number restored to good Sight by Operation* | *Restored to a degree of useful Sight by Operation* | *Total Number of Cataracts, Artificial Pupils, and Pterygiums, successfully treated by Operation.* |
|---|---|---|---|---|---|
| Cataracts ...... | 407 | 29 | 436 | 43 | 479 |
| Artificial Pupils | 9 | .. | .. | 3 | 9 |
| Pterygiums .... | 2 | .. | .. | .. | 2 |
| Total successfully treated by surgical operations ............. | | | | | 490 |
| Restored to good sight by medical treatment .............. | | | | | 14 |
| General total restored to sight ............................. | | | | | 504 |

By referring to the table of diseases, it will be perceived that, in the course of seven months and twelve days, 479 cases of cataract have been successfully treated; nine cases of closed pupil, and two of pterygium, have been equally so by operations; and fourteen with diseased cornea have received good sight by medical treatment; which, when added together, will make a total number of 504 blind restored to sight.

A great number of these patients, on whom the operation for cataract has been performed, can read the figures of a watch without the aid of glasses; a medium which would nearly perfect their sight, if assisted by them.†

Those who are stated in the column of diseases to enjoy but a small degree of vision, though already highly useful to them, would also be greatly benefited by the assistance of these glasses; because in most of them the imperfection of sight is owing to the flatness of the cornea dependant on old age.

Twenty-nine having lost their sight after it had been restored to them by operation, was caused by the patients not being

---

* We think it proper to state, that the Report containing these observations is countersigned by James French, m.d. assistant surgeon 67th Regiment, and Charles Ducat, m.d. civil surgeon, Poonah.—Editors.

† These glasses can be purchased in England for a shilling a pair.

under restraint, nor willing to submit to any kind of medical control. Many who received sight in one instant rejoiced so exceedingly, that they became impatient of remaining a few days with their eyes bound up; and after they had left me, to enjoy the pleasure of vision and the sight of their friends, uncovered their eyes, and admitted the strong beams of light on the retina, which had been for years secluded in darkness, and was consequently unable to withstand the first impressions of light without exciting inflammation. On comparing this loss with the general success of operations for cataract, it will be found that it has occurred in about one in twenty.

Though I have not the least reason to complain of the want of confidence of the natives, yet I find them unwilling to submit, after treatment, to any painful remedy; for, had these people subjected themselves to the ordinary means employed in averting inflammation, I am certain their sight would have been preserved.

The disadvantageous mode of operating on the natives of India, militates materially against the propitious result of practice; for, after the operation is performed, they are allowed to go wherever they please, and follow their own inclinations. But, to overcome these inconveniences, and to prevent succeeding inflammation as much as possible, the eye undergoes very little disturbance during the operation; so that in most cases no after treatment is required, except the occasional application of a few leeches to the temples.

To manage the natives with readiness, it is absolutely necessary to do much with one stroke of the instrument; light must be given in one instant, and with as little pain as possible. This can always be done with a hard cataract; but one that is soft, and will not bear the pressure of the needle, requires some time to be removed from the axis of vision. I, therefore, always apprise the patient of this probable incident.

I have many times passed a needle through the walls and humours of the eyeball, without the patients showing the least symptom of pain. They remained as firm and steady as if nothing of the kind had taken place; and, when questioned regarding the degree of pain, some answered they felt none, others felt a little. I am, therefore, of opinion that, with a highly-polished instrument, the operation of couching may be always performed, and the cataract laid down below the axis of vision, so as never to rise, without the patient feeling any more pain than that of blood-letting, and frequently not so much.

During the time I have been in Poona, the number of applicants amounts to 820, many of whom have been saved from blindness by the timely interference of medical treatment. About 100 people with incipient cataract have also applied for relief, but, not being completely blind, I could not think of proposing an operation, but informed them that a total deprivation of sight would most probably ensue in the course of eighteen months or two years, and the eye would then be in a proper state to receive assistance by operation. I have not noted down any of these people's names, because they did not actually come under medical treatment. Adding all these to the number last mentioned, will make 920 applicants.

A few weeks ago, I went down to the river's side, where an old blind woman resided: the structure of her left eye was totally destroyed, but the right contained a fine cataract, which was removed in an instant, and sight restored. In less than half an hour after this operation, a crowd of lame and blind surrounded me, among whom I found ten were blind with cataract. On them I continued operating on the bank of the river till it grew dark, when I found I had but operated on eight, of whom seven had received good sight: the other one did not derive any benefit, on account of the principal nerve of vision being diseased. I then returned home, leaving two for operation, who followed me the next day, and received sight.

On another day, in presence of two gentlemen, by operation I restored fourteen out of fifteen to good sight; and on another day I went a course of forty miles, to Sassoor, and villages adjacent to it, in company with Drs. French and Ducat, who very kindly assisted me to perform twenty-eight operations, out of which, twenty-seven proved successful; and within the space of two days, with the same gentlemen, I rode a course of fifty miles, to Telligahum and villages lying round it, where I operated with success on twenty-seven cases of cataract.

Many of these patients included in this abstract came from the neighbouring villages of Poonah, and some from the distance of 150 miles.

***Abstract of Diseases of the Eye, treated at Ahmednuggur by Surgical Operations, from the 8th of January to the 8th of March, 1825.***

| *Diseases.* | *Restored to good Sight by Operation* | *Restored to good Sight by Operation; but, by the imprudence of the Patient, Inflammation was brought on, which destroyed the Eyes.* | *Total Number restored to good Sight by Operation* | *Restored to a degree of useful Sight by Operation* | *Total Nnmber of Cataracts and Artificial Pupils successfully treated by Operation.* |
|---|---|---|---|---|---|
| Cataracts ...... | 205 | 4 | 209 | 13 | 222 |
| Artificial Pupils | .. | .. | .. | 4 | 4 |
| Total successfully treated........................... | | | | | 226 |

In the course of the first month after my arrival here, I restored sixty-six blind to sight; and in the course of the second, 156; making 222 successful operations of couching and extraction of cataracts: 150 of these were found in the town, and seventy-two in the neighbouring villages. Thirteen received sight only to a partial degree, but to such extent as to render them essential service.

Four men, who were blind from closure of the pupils, were restored to a degree of sight very conducive to the common purposes of life. These, added to the number above, make a total of 226 blind restored to sight in the space of two months. One hundred and two, with other diseases of the eye, were at the same time either cured or relieved; which raises the number of applicants to 328.

One boy, six years of age, who had been in a state of blindness four years, arising from cataract, received sight by the operation. Another boy, twelve years of age, who had been blind ten years with the same disease, was also restored to sight by the operation.

From the imperfect account which the parents and the children gave me of the date and origin of their blindness, I am strongly inclined to infer that they were born blind with cataract: although they got good sight, they did not know the name of any thing until they learned it. In support of this opinion, I beg to mention that, when I was at Poona, there was brought to me a boy, five years of age, who was said to have been blind three years with cataract. A few days after the operation he recovered sight, but two months elapsed before he learned the names of common things around him, and understand the meaning of the term vision. Sometimes he appeared to be more stupid and awkward than when he was blind. In consequence of these circumstances, his parents concluded I had done him no good: I was convinced, however, that he saw well; and it was not long till he became a fine active boy, and capable of discerning very small objects. This happy restoration to sight, as he was heir to a large property, was the more eagerly desired by his parents.

By comparing all the circumstances in the cases of these three boys, it will be observed, that blindness was not perceived in them till they began to walk, and probably to stumble, when the attention of the parents was arrested, and drawn into the examination of the causes. In the case of all the children, the near coincidence of time from their birth to the time of their supposed bereavement of sight, is striking, and to me conclusive that they were born blind: for the parents of the two boys agree in reporting that they did not perceive the want of sight in the children till they were about two years of age; and the mother of the boy who was ten years blind said, that she did not perceive his loss of sight until he had arrived at the age of eighteen months.

In contrasting these cases with the following one, the subject will be still further illustrated. A boy, twelve years of age, who had been blind eight years of one eye, from closure of the pupil, and had the structure of the other destroyed. The moment the new pupil was cut out, he uttered with vehemence that he saw much light, and became so overjoyed, that his parents could not restrain him within the walls of the hospital. He romped through the house in such a manner, and examined every article so minutely with his hand and eye, as to raise suspicions of his honesty. At the end of five days, he left the place, without the least stigma on his character.

When this boy was deprived of sight, he was double the age of any of the other three, and he therefore became acquainted with his native language. The recollection of things which he had seen eight years before was not entirely effaced from his memory, so that, after he received sight, the presentation of these things recalled the remembrance of them. But, owing to the early deprivation of sight, and the long space of time which elapsed while in that state, the muscles of the eye not having been called to act in the various degrees of compressing and steadying the globe, could not at first adjust the focal point, and required some time for that purpose, though the lens was left in perfect health. But in the other boys, who had been deprived of sight at an earlier period of life, and had their lenses removed, the motions of the eyeball were strong and involuntary, requiring some weeks before each individual muscle was brought under its own function, in fixing the eye on an object.

I hope to be pardoned for the digression I have made from the tenor of my Report; but the subject of congenital cataract is so interesting, and especially the extent to which it prevails among the natives, that I could not think of letting this opportunity pass without notice.

Several times I travelled thirty-five miles a-day among the neighbouring villages, and in the course of the day restored twenty blind to sight. On three other excursive days, I restored fifty-two to sight. I went on one occasion into Jamghaum, a village belonging to Scinda, where I restored eight blind to sight, and received great civility from the inhabitants.

Those patients who resided at a considerable distance from Ahmednuggur, I did not revisit till sixteen days after the operations, when I found some of them, without any person to conduct them, traversing the fields, and visiting their neighbours in different parts of the villages. This space of time, which had been allowed to elapse from the date of my first visit, showed the result of the operations, and enabled me to give a correct account of their success.

Although these patients were left entirely to the natural consequence of the operations, without medicine or the application of a single leech to the temples, and exposed to the baneful effects of a tropical climate, only two eyes were lost by inflammation in the villages, and two in the town of Ahmednuggur; so that the proportion of losses is one in fifty-four.

On my return to these patients, I found many whose eyes were without the least redness, pain, or symptom of having been operated on, at so late a period. Some of them conveyed me out of the villages, and held me by the coat, until they made me acquainted with a sense of their heartfelt gratitude. The favourable termination of these cases, I beg to mention as a proof of the extreme mildness of the operations. With respect to soft and fluid cataracts, I have been able to dispose of their substance in such a manner, that the eye cleared up in sixteen days, and enabled me to report on them with accuracy.

I find almost all classes of people equally liable to cataract, and, if any difference exists, I am inclined to think that it prevails most among the peasants, artisans, and coolies, because these people are greatly exposed to the causes of cataract, and are compelled to earn their bread under the baneful effects of the climate. In villages, I find as many cataracts, proportionally to the number of inhabitants, as in large towns. Occasionally, when I find twenty or thirty people together on the road, I ask leave to look at their eyes, and not unfrequently I find in the group one or two eyes blind with cataract, which I immediately remove, and send them away with instructions to call on me.

In the case of some who had undergone operation for cataract, I found the common spectacles, ground so as to throw the focus a considerable way back, so far supply the place of cataractous glasses in improving vision, as to enable them to read their native language.

In answer to a letter of the 7th December, 1824, which I had the honour to receive from the Honourable the Governor in Council, through the Medical Board, directing me to turn my attention, more particularly than I had done, to the instructing of native practitioners in the knowledge of diseases of the eye, and the method of performing the various operations on it for the recovery of sight; I have the honour to state, that I lost no time in endeavouring to carry these instructions into effect.

I informed the native practitioners that I had received orders from government to teach them every part of this branch of the profession; that the great aim of government was to ameliorate their condition, by diffusing knowledge among them. I pointed out to them how much advantage it would be to themselves to learn to restore sight without risk of destroying the eye; and this was to be accomplished in no other way than by studying the structure of the organ, with which I would make them acquainted, if they would favour me with their daily attendance; and would also teach them an easy and ready method of removing cataract.

Accordingly, eight Mussulman practitioners regularly attended me; and, on those days on which they anticipated operations, they were punctual to the appointed time, and seemed much pleased when I employed them as assistants. After they had been with me a few days, they manifested a strong desire to be made acquainted with the principles of the operations; and they appeared much gratified at the simplicity of couching.

The power of belladonna in enlarging the compass of the pupil, and giving a full view of the cataract, and thus showing the operator, when the needle was in the eye, so to direct its point as not to lacerate the structure within the organ, excited their admiration.

I frequently demonstrated the structure of the eye to them, and taught them the site of the lens which, in consequence of disease or injury, formed cataract; but this they appeared to have some difficulty in understanding, until I extracted a number of cataracts, and laid them on their hands, when they examined the form and substance of them, and were much pleased at the disease being extracted. This step was the best I had yet found for giving them a correct idea of the subject. They were always ready to assist me, and willing to learn, but lamented their utter inability to procure instruments and belladonna. Had my continuance been longer in the place, I have no doubt I should have been able to carry on their knowledge, for they often frequented my lodgings for the purpose of being taught.

The instruments with which they operate is an old rusty lancet, with the point broken off, and coarsely ground to an oval form: with it they pierce, or rather lacerate, the coats of the lower hemisphere of the globule, behind the iris; then pass a triangled copper probe through the lacerated orifice into the eye, and endeavour to depress the cataract.

In old people, where the vitreous cells are obliterated, and there is no adhesion between the lens and its capsule, they succeed in one in seven, or one in eight cases. But, where a fortuitous attachment between the lens and its capsule has been confirmed by disease, and the vitreous cells remain unbroken, the prospect of success, with instruments like theirs, whoever he be who uses them, must be feeble. I have made this calculation from cases that came before me, and must say, that, if they have been so fortunate as not to commit mischief, by bringing on insuperable inflammation, and destroying the structure of the eye, they have at least done so much injury as to bring on amaurosis, and prevent the image from being depicted on the retina.

Since writing this Report, one of the Mussulman practitioners mentioned in the preceding part of it, has just returned from a distance of twenty-four miles, and informed me that he there restored five blind to sight, with an old needle which I gave him.

### APPENDIX.

| | |
|---|---|
| The city of Poona is supposed to contain 100,000 inhabitants; the number of blind restored to sight there was . . . . . . | 504 |
| The town of Ahmednuggur is supposed to contain 20,000 inhabitants; the number of blind restored to sight there was . . | 226 |
| General total restored to sight in the course of nine months . | 730 |

| | |
|---|---|
| Cataracts in the above number removed with success . . . . | 701 |
| The number of artificial pupils made with success . . . . . | 13 |
| Pteryguims removed . . . . . . . . . . . . . . . . | 2 |
| Number of blind restored to sight by medical treatment . . | 14 |
| General total of blind restored to sight . . . . . . . . . . | 730 |

On comparing the size of these towns with those in Europe, I am of opinion Poona contains only 80,000 inhabitants, and Ahmednuggur 15,000.

